# Use of the bolus tracking technique for the tomographic evaluation of the uretero-vesicular junction in dogs and assessment of dose records

**DOI:** 10.1186/s12917-016-0690-z

**Published:** 2016-03-29

**Authors:** Maurizio Longo, Maria Elena Andreis, Cinzia Pettinato, Giuliano Ravasio, Vanessa Rabbogliatti, Donatella De Zani, Mauro Di Giancamillo, Davide Danilo Zani

**Affiliations:** 1 Department of Veterinary Medicine (DIMEVET), Università degli Studi di Milano, Ospedale Didattico Universitario Az. Polo Veterinario di Lodi, Via dell’Università 6, 26900 Lodi, LO Italy; 2 Medical Physics Unit, S.Orsola-Malpighi University Hospital, Bologna, Italy

**Keywords:** uro-CT, uretero-vesicular junction, bolus tracking, DLP, CTDI, ureter

## Abstract

**Background:**

The aim of the work is the application of a bolus tracking technique for tomographic evaluation of the uretero-vesicular junction in dogs. Ten adult dogs (8–14 years) with variable body weight (2,8–32 kg) were enrolled in the prospective study. The patients were placed in sternal recumbency with a 10° elevated pelvis and the visualization of the uretero-vesicular junction was obtained with the bolus tracking technique after intravenous administration of non-ionic contrast medium. In the post-contrast late phase a region of interest was placed within the lumen of the distal ureters and the density values were monitored before starting the helical scan.

**Results:**

The uretero-vesicular junction was clearly visible in 100 % of patients with the visualization of the endoluminal ureteral contrast enhancement and bladder washout. At the end of the tomographic study an evaluation of the dose records was performed and compared to human exposures reported in literature for the pelvic region. The effective dose estimated for each patient (37,5–138 mSv) proved to be elevated, when compared to those reported in human patients.

**Conclusion:**

The bolus tracking technique could be applied for the visualization of the uretero-vesicular junction in non-pathological patients, placing the region of interest in the distal ureters. The high effective doses recorded in our study support the need of specific thresholds for veterinary patients, pointing out the attention for paediatric patient’s exposure also in veterinary imaging.

## Background

Ureteral ectopia is a pathological condition that occurs as a result of abnormal caudal migration of the ureteral bud at the level of its insertion to the urinary bladder [1]. This pathological condition, widely represented both in humans and animals, can lead to severe mechanical obstruction with consequent hydroureter, hydronephrosis and pyelectasis in chronic and more severe stages of the disease. Computed tomography has been considered the modality of choice for the evaluation of the pelvic region, avoiding superimposition of structures, limited contrast and spatial resolution. Computed tomography Excretory Urography (CTEU), dynamic scans of the pelvic region, has been reported [2–8] as a useful tool to evaluate the distal ureteral jet of the contrast media. Moreover, the administration of complementary drugs, such as furosemide, or different patients positioning, supine vs sternal, for the optimization of the visualization of the distal portion of the ureters has been well investigated [7–12].

Because of the difficulty to correctly visualise the uretero-vesicular junction, very often many repeated helical CT scans of the pelvic region are required, strongly increasing patient exposure. In order to achieve qualitative images of the uretero-vesicular junction, optimizing scanning time delay is mandatory, although it could be widely different among patients [7, 8].

The aim of this work was to evaluate the feasibility to use the bolus tracking technique to visualize the uretero-vesicular junction in dogs. Additionally, an estimation of the exposure parameters was performed.

## Methods

Dogs referred to our Institution for neoplasms staging and without signs of urinary tract illness have been enrolled in the prospective study. Premedication was not standardized. In all dogs, anaesthesia was induced with propofol and maintained by isoflurane in oxygen 100 % (Table 1).

**Table 1:** Breed, Weight, Age, Sex, CT indication and Anaesthetic Protocols

Breed	Weight (kg)	Age (years)	Sex	CT indication	Anaesthetic protocol
Akita	30	13	NF	rectal neoplasm	I: dex 3 μg/kg e.v. + ppf ∼ 2,5 mg/kg e.v.
					M: iso in oxygen 100 %
Pinscher	6	14	IM	bone neoplasm	I: dex 3 μg/kg e.v. + ppf ∼ 2,5 mg/kg e.v
					M: iso in oxygen 100 %
Mixed breed	5,5	8	IM	suspected hepatic neoplasm	I: dex 3 μg/kg e.v. + ppf ∼ 2,5 mg/kg e.v.
					M: iso in oxygen 100 %
Irish Setter	26	8	NF	mammary neoplasm	I: dex 3 μg/kg e.v. + ppf ∼ 2,5 mg/kg e.v
					M: iso in oxygen 100 %
Labrador Retriever	32	10	NF	hepatic/splenic neoplasm	P: dex 1 μg/kg e.v + but 0,2 mg/kg e.v.
					I: ppf e.v. to effect (∼ 4 mg/kg)
					M: iso in oxygen 100 %
Pinscher	5	8	IF	mammary neoplasm	P: dex 5 μg/kg i.m. + met 0,2 mg/kg i.m.
					I: ppf ∼ 2,5 mg/kg e.v
					M: iso in oxygen 100 %
Mixed breed	30	11	IF	mammary neoplasm	P: dex 5 μg/kg i.m. + met 0,2 mg/kg i.m.
					I: ppf e.v. to effect (∼ 4 mg/kg)
					M: iso in oxygen 100 %
Pinscher	2,8	8	IF	mammary neoplasm	P: dex 10 μg/kg i.m. + but 0,2 mg/kg i.m.
					I: ppf e.v. to effect (∼ 4 mg/kg)
					M: iso in oxygen 100 %
Pug	11	12	IM	mandibular neoplasm	P: but 0,2 mg/kg e.v.
					I: ppf e.v. to effect (∼ 4 mg/kg)
					M: iso in oxygen 100 %
English Setter	20	10	IF	mammary neoplasm	P: dex 5 μg/kg i.m. + met 0,2 mg/kg i.m.
					I: ppf e.v. to effect (∼ 4 mg/kg)
					M: iso in oxygen 100 %

*NF* neutered female, *IM* intact male, *IF* intact female, *P* premedication, *I* induction, *M* maintenance, *ppf* propofol, *dex* dexmedetomidine, *iso* isoflurane, *but* butorphanol, *met* methadone

Images were acquired using a 16-slices CT scanner (GE Brightspeed®, GE Healthcare Milano - Italy). Dogs were examined in sternal recumbency with the pelvis elevated on a wedge with a 5° to 10° angle [13]. CT acquisition parameters, according to patient dimensions, were set as follows: 120 kV, 150–200 mA, 1,25 mm thick contiguous slices.

For contrast enhanced CT images, a bolus injection of a non-ionic iodinated contrast medium, Ioexhol (Omnipaque® 350 mg/ml, GE Healthcare Milano - Italy) was administered into the cephalic vein via 18–27 G catheter at a dose of 600mgI/kg, using a pressure injector (Medrad® Mark V Plus, Milano - Italy) and a rate of 2–3 ml/s.

The acquisition protocol was as follows: 1) total body CT scan; 2) contrast enhanced total body CT scan; 3) post contrast late phase limited to the abdomen, using the bolus tracking on the ureters.

**Fig. 1:**
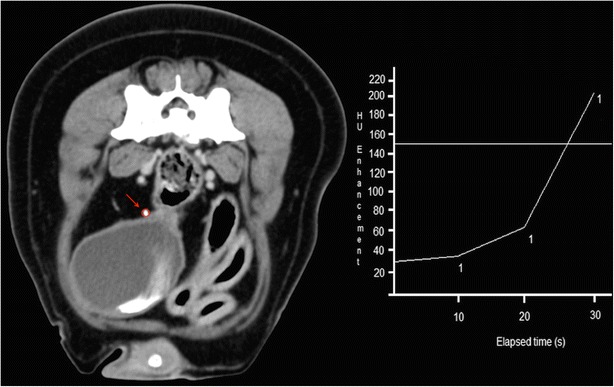
Transverse image at the level of the distal third of the right ureteral lumen, cranial to the caudo-medial deflection. Positioning of the ROI (*red arrow* and *circle*) and tracking of the progressive intraluminal peak enhancement (HU enhancement/elapsed time diagram)

Contrast medium was injected into the patient and a ROI was positioned on the distal third of the ureteral lumen, cranial to the caudo-medial deflection, in order to track the volume of contrast (Fig. 1). CT images were acquired, according to literature_,_ after the 150 HU level was reached into the ROI [14]. During the monitoring phase, low exposure (50 mA) repeated axial scans were performed at the pre-selected level (Fig. 1). By means of a built-in software program a near instantaneous time attenuation curve was generated. When contrast medium reached the desired 150 HU (Fig. 1) the operator triggered a complete helical scan of the caudal abdomen.

Lactated Ringer’s solution was infused during anaesthesia at a rate of 5 ml/kg/h to prevent contrast medium induced acute kidney injury (CI-AKI) [15, 16].

Dose reports were stored for each acquired CT image series. Images have been evaluated using a certified software 64 bit OsirixMED® (Aycan Medical System, LLC) and the identification of the uretero-vesicular junction was assessed for each patient in transverse slices, dorsal and longitudinal reconstructions (Volume Rendering, Multiplanar Reformation and Maximum Intensity Projection).

The scans of interest for the evaluation of the ureteral junction (conventional unenhanced and enhanced series, low exposure axial series and late enhanced series with bolus tracking technique) were selected from the Dose Report of each dog. The Dose Length Product (DLP) values for each scan were added together in order to obtain an estimated total DLP value for each dog. Using the formulas suggested in the AAPM 96 report [17] the effective dose for each scan was calculated by multiplying the DLP by the corrected conversion factor. In particular conversion factors of different human sizes (0 year old, 1 year old, 5 years old, 10 years old and adult) based on patient weight were used (Table 2).

**Table 2:** CTDI Vol, Partial and Total DLP, DPL Refined with Human Pelvis Weighing Factor

Breed	Weight (kg) (reference man)	Scan	CTDI vol (mGy)	DLP	Human pelvis weighing factor	Effective dose (mSv)
				(mGy-cm)		
Akita	30 (10 yo)	CU	19.93	2002,77	0,015	30,0
		CE	19.93	2002,77		30,0
		SA	29.17	29,17		0,4
		LEBT	19.93	906,5		13,6
				4941,21		74,1
Pinscher	6 (1 yo)	CU	22.30	889,9	0,03	26,7
		CE	22.30	889,9		26,7
		SA	66.67	66,67		2,0
		LEBT	22.30	103,8		3,1
				1950,27		58,5
Mixed breed	5.5 (1 yo)	CU	18.94	894,31	0,03	26,8
		CE	18.94	894,31		26,8
		SA	45.84	45,84		1,4
		LEBT	18.94	894,31		26,8
				2728,77		81,9
Irish Setter	26 (10 yo)	CU	19.93	1305,14	0,015	19,6
		CE	19.93	709,67		10,6
		SA	29.17	29,17		0,4
		LEBT	19.93	458,02		6,9
				2502		37,5
Labrador Retriever	31 (10 yo)	CU	21.71	2200,23	0,015	33,0
		CE	21.7	2200,23		33,0
		SA	33.34	33,34		0,5
		LEBT	19.93	874,11		13,1
				5307,91		79,6
Pinscher	5 (1 yo)	CU	19.93	799,36	0,03	24,0
		CE	19.93	799,36		24,0
		SA	12.50	12,5		0,4
		LEBT	19.93	799,36		24,0
				2410,58		72,3
Mixed breed	30 (10 yo)	CU	19.93	1397,33	0,015	21,0
		CE	19.93	1397,33		21,0
		SA	12.50	12,5		0,2
		SA	12.50	29,17		0,4
		LEBT	19.93	1397,33		21,0
				4233,66		63,5
Pinscher	2,8 (0 yo)	CU	17.94	930,21	0,049	45,6
		CE	17.94	930,21		45,6
		SA	25.00	25		1,2
		LEBT	17.94	930,21		45,6
				2816,63		138,0
Pug	11 (5 yo)	CU	19.93	1193,02	0,02	23,9
		CE	19.93	1193,02		23,9
		SA	12.50	12,5		0,3
		LEBT	19.93	1193,02		23,9
				3591,56		71,8
English Setter	20 (5 yo)	CU	19.93	1153,16	0,02	23,1
		CE	19.93	1153,16		23,1
		SA	54.17	54,17		1,1
		SA	25.00	25		0,5
		LEBT	19.93	1153,16		23,1
				3538,65		70,8

*NF* neutered female, *IM* intact male, *IF* intact female, *CU* conventional unenhanced, *CE* conventional enhanced, *SA* single axial, *LEBT* late enhanced with bolus tracking technique

## Results

Ten dogs of different breeds (4 intact females, 3 neutered females, 3 intact male) were enrolled. Body weight varied from 2,8 to 32 kg (mean 16,83 kg) and the mean age was 10,2 years, ranging from 8 to 14 years. All dogs had no history or signs of urinary tract illness, based on blood tests and pre-anaesthetic clinical evaluation.

**Fig. 2:**
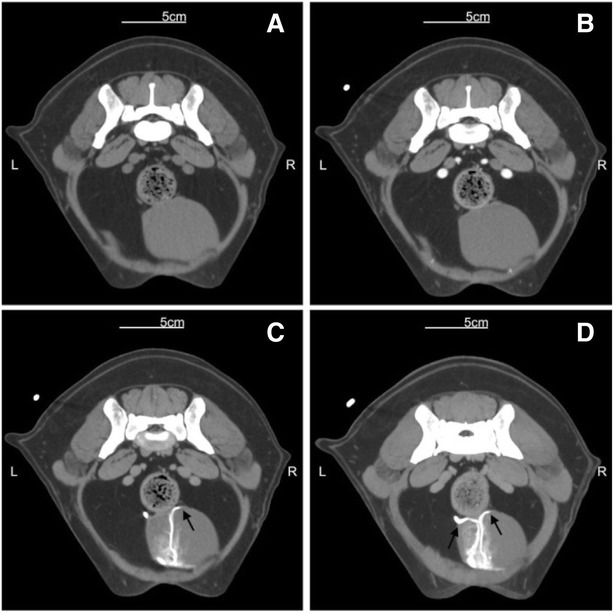
Transverse images at the level of the uretero-vesicular junction. Gradual distribution of the contrast media: unhenhanced conventional scan (**a**), vascluar distribution in the enhanced conventional scan (**b**), late post-contrast enhanced series with bolus tracking technique (**c**), reconstructed by MIP (**d**). Black arrows indicate the uretero-vesicular junction

**Fig. 3:**
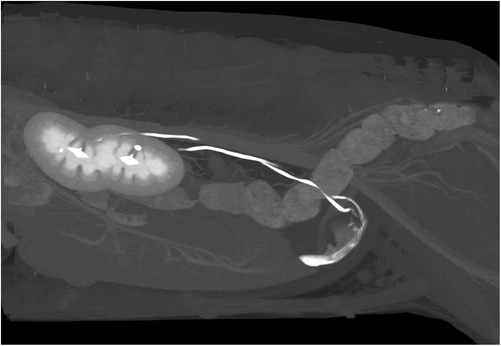
Post-contrast sagittal 3D MIP of the abdomen showing the enhanced uretero-vesicular junctions and the ureteral washout

The uretero-vesicular junction, was easily identified in all (100 %) patients in the late bolus tracking scans, and clearly visualized when the MIP post-processing technique was adopted (Figs. 2 and 3).

Based on Dose reports the volumetric computed tomography dose index (CTDI vol) values, both partial and total dose length product (DLP) values and effective doses were estimated (Table 2). The effective dose range was quite wide due to different patient weights. In particular the average effective dose for the pelvic region acquisition was 20.1 ± 11.9 mSv (range 3.1–45.6 mSv) and average effective dose, considering the whole protocol, was 74.8 ± 25.6 mSv (range 37.5–138 mSv).

## Discussion

The bolus tracking technique is a tomographic modality of bolus contrast synchronization widely applied in human medicine, mainly for the examination of the cardio-circulatory system and perfusion index of neoplasms, especially located in the liver [18, 19]. Bolus tracking technique allows a real time monitoring of the contrast bolus by selecting a region of interest commonly positioned in the lumen of a vessel [18]. To the author's knowledge, the visualization of the uretero-vesicular junction by placing a ROI into the ureteral lumen has never been reported before.

The need to apply this dynamic scan technique to the urinary tract has been inspired by the everyday time-consuming radiographic and tomographic examination of the excretory apparatus in veterinary patients, as in case of CTEU or combined intravenous pyelogram (IVP) and CT. This kind of studies can be strongly influenced by many variables, such as patient dimensions, body weight, total blood volume, heart rate and contrast medium [20]. These parameters, highly variable in physiological conditions, may differ even more when pathological conditions occur, such as ureteral ectopia, and when anaesthetic drugs are administered [21, 22].

In this study the bolus tracking technique has been applied in dogs with no signs of urinary tract illness, in order to verify its application on this apparatus in normal conditions. The uretero-vesicular junction was recognisable as a hook-shaped structure corresponding to the medial deflection of the distal ureters as they insert in the bladder wall [23]. It was easily identified in all patients in the late bolus tracking scans and better visualized when the MIP post-processing technique was used. Some authors indicated coronal views for humans as the best reconstructions to allow a successful visualization of the distal ureters without MIP, since this technique is reported to reduce the density values of the enhanced ureters in humans [3]. Our results are partially in agreement with those previously reported: we observed a slight enhancement reduction using MIP but this did not influence the ability of the radiologist in the thorough visualization of the uretero-vesicular junction. This could probably lead back to the anatomical difference of the abdominal anatomical shape between humans and animals, more compressed dorso-ventrally in non-obese people than in animals, where it usually develops in a circular transverse shape. For this reasons it could be easier to obtain a coronal reconstruction of the caudal abdomen in humans, for a correct estimation of the uretero-vesicular junction because in the same plane coronal images can provide the appearance of both kidneys and ureters.

The application of the bolus tracking technique by selecting a ROI in the ureteral lumen allows bypassing most of the above-quoted issues, clearly visualizing the uretero-vesicular junction, in normal conditions. Moreover, our study shows that the bolus tracking technique can be successfully applied despite different anaesthetic protocols as the uretero-vesicular junction is clearly visualized. Further studies will be required in the future to evaluate the applicability of the bolus tracking technique in patients with urinary tract illness and after the administration of other pre-anesthetic and anesthetic drugs.

An additional pressing problem, as far as the radiographic and tomographic exam of the pelvis is concerned, could be patient exposure [4, 5]. X-rays are accountable for both deterministic and stochastic effects [24–26] and precise thresholds are available for humans [25]. Many authors [4, 5, 9] evaluated human patients exposure during CT pelvic exams, but no evaluation has ever been proposed for veterinary patients: this was the second aim of this study. Patients exposure was estimated choosing the appropriate scan series in the dose report of each patient, and later refining them with the application of human pelvis weighing factors (Table 2). The estimation of the effective doses to pelvis turned out to be quite elevated if compared to human reported values [2–8] but it must be taken into account that these values could be affected by the weighting factors related to human references and that the acquisition protocol parameters are not optimized for dose reduction purposes. However, the effective doses we estimated were in any case well away from deterministic human damage thresholds and never got closed to stochastic damage thresholds either. Furthermore, the aim of this study was also to demonstrate that the use of bolus tracking could decrease patient exposure. Actually, the ability of this technique to identify the optimal time delay for ureters visualization, allows the reduction of patient dose by avoiding useless exposures due to repeated acquisitions.

## Conclusions

We consider the bolus tracking technique as a useful tool to evaluate the distal portion of the ureters and the uretero-vesicular junction in dogs without urinary tract illness. This modality can enhance the visualization of this region, possibly limiting the effective dose the patients receive from conventional uro-tomographic studies of the pelvis. Even if radio-exposure could be a minor concern in veterinary patients, when compared to humans, we would like to recommend a stressful attention to radio-exposure for this type of studies, not only for the anatomical region exposed, but also for the high prevalence of paediatric veterinary patients admitted for this exams [26]. Future perspectives will be oriented to the application of ureteral bolus tracking technique in pathological conditions, taking into consideration the reduction of the effective doses for veterinary patients.

### Declarations

The study have been performed under written informed consent of the owner in each case, according to Animal ethics national/EU laws.

## Abbreviations

CT: computed tomography
CTDI vol: volumetric computed tomography dose index
CTEU: Computed Tomography Excretory Urography
DLP: Dose Length Product
IVP: intravenous pyelogram
MIP: Maximum Intensity Projection
ROI: region of interest
